# Analysis of swallowing in infants and adults using speckle pattern analysis

**DOI:** 10.1038/s41598-022-07895-w

**Published:** 2022-03-09

**Authors:** Raz Shahmoon, Yitav Tamir, Yevgeny Beiderman, Sergey Agdarov, Yafim Beiderman, Zeev Zalevsky

**Affiliations:** Bar-Ilan University, Ramat Gan, Israel

**Keywords:** Health care, Optics and photonics

## Abstract

The ability to detect and evaluate ingestion is especially important in toddlers. The development of new methods for detecting and accurately measuring ingestion is therefore extremely important. One of the methods allowing such measurements is speckle pattern analyses, a well-known phenomenon in coherent imaging. The method allows extraction of various medical parameters, such as blood pulse pressure, temporal signature of heartbeats and breath. The current work contains further development and application of the speckle tracking technique for remote detection and quantification of swallowing and distinguishing between sucking and swallowing to identify feeding disorders in infants and elderly individuals.

## Introduction

### The swallowing process

**Figure 1:**
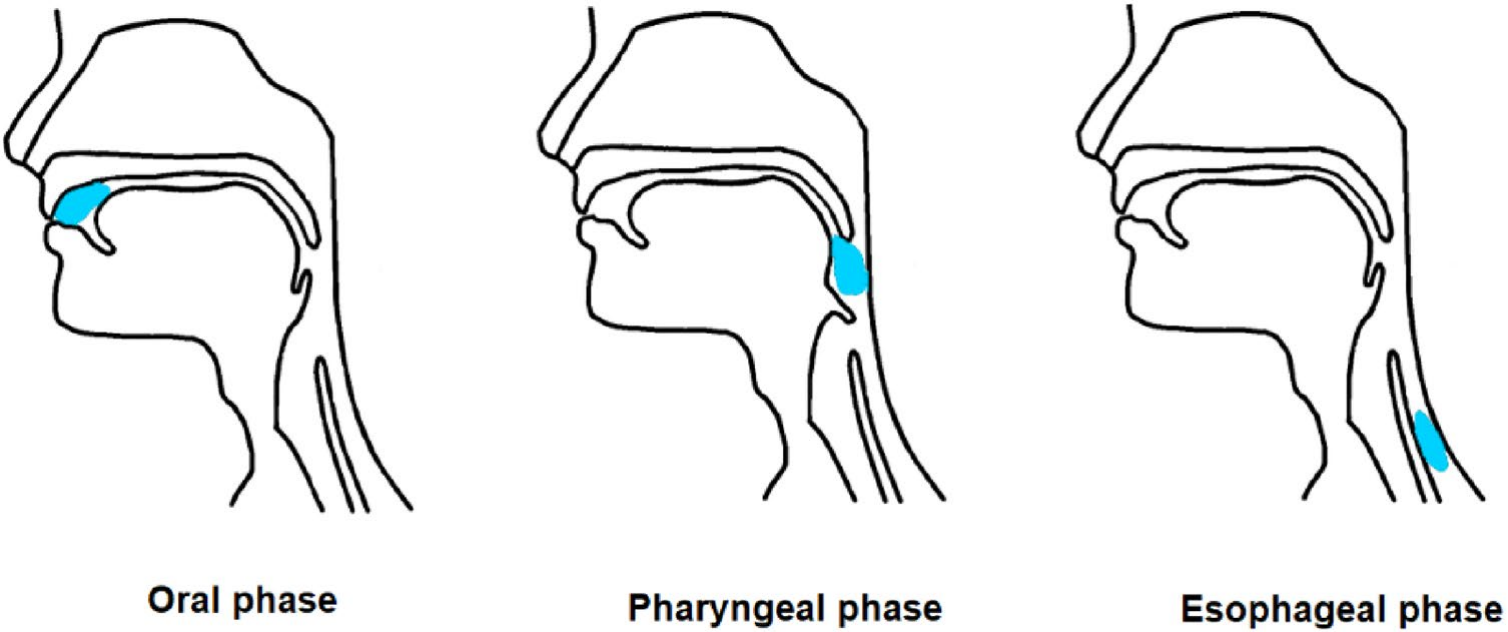
The three phases of swallowing.

Swallowing is an important part of eating and drinking, being a complex act consisting of three phases, the oral, pharyngeal and esophageal phases (see Fig. 1). With a single sip of liquid, the pharyngeal phase follows immediately. For swallowing solid food, there may be a delay of 5 or 10 s^1^.

**Figure 2:**
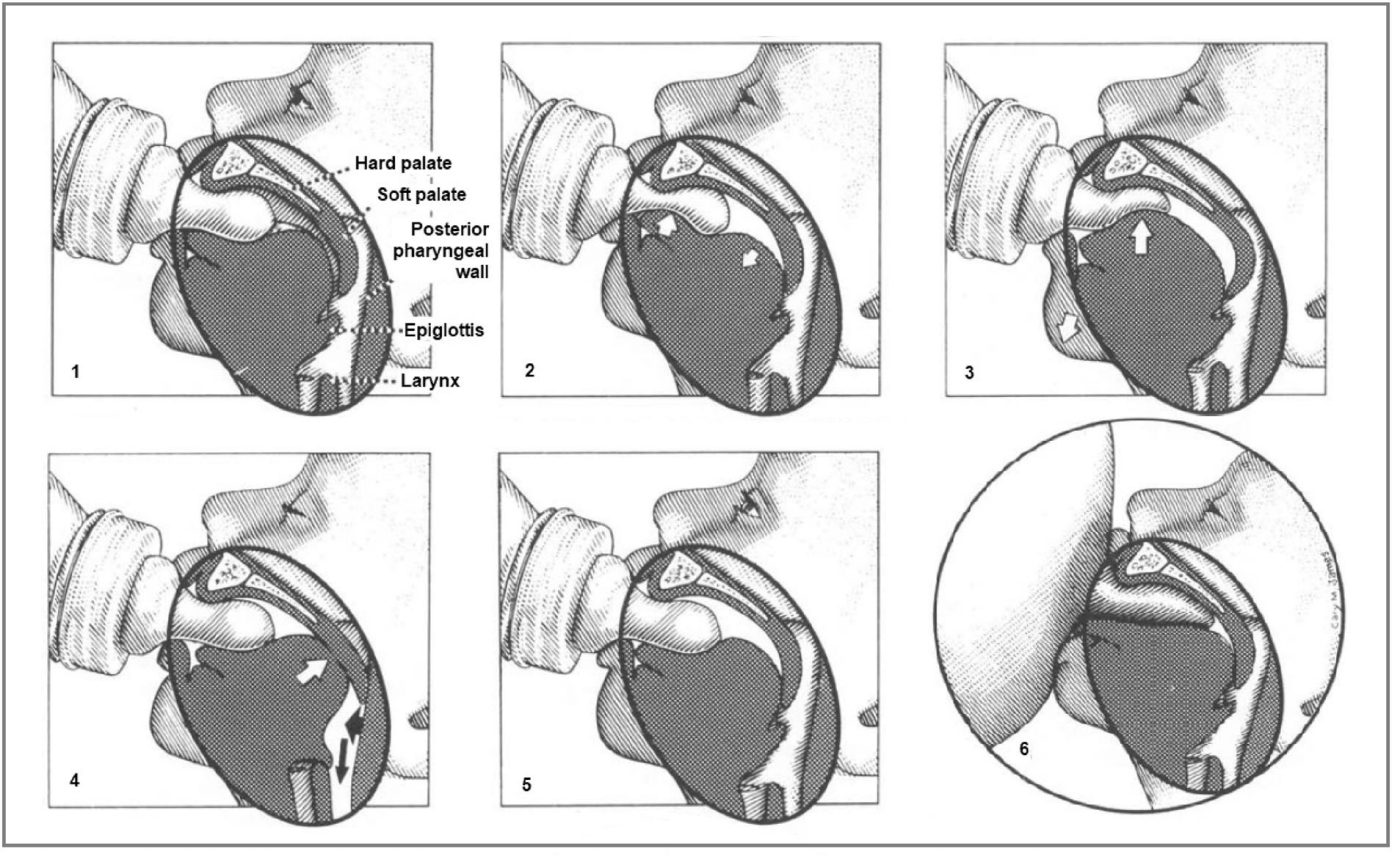
Sucking and swallowing stages among infants (The picture is a copy from the Ref.^3^).

The swallowing process among infants is more complex. Sucking and swallowing are functions vital to a newborn infant. The sucking movement precedes swallowing, which in turn inhibits respiration. Therefore, efficient and safe feeding requires coordination of breathing with sucking and swallowing and involves the functional interaction of the lips, jaw, tongue, palate, pharynx, larynx and esophagus, as presented in Fig. 2. Together, these functions are responsible for the swift and safe transport of milk boluses from the oral cavity to the stomach^2–4^.

Sucking occurs when an infant begins to nurse. The infant’s nursing dynamic should involve one to two vigorous and rhythmical sucks to one swallow. The most efficient milk transfer is associated with a suck-to-swallow ratio of 1:1 or 2:1. Ratios of 5:1 or higher are associated with piston movements of the infant jaw and poor weight gain. Nonnutritive sucking, in case it is systematic and affects an infant, could be considered a disorder. Therefore, remote detection of irregularity in the sucking–swallowing composition could indicate the existence of a feeding problem before it affects an infant. Swallowing and feeding disorders in infants and children are complex and could have multiple causes in various categories of disorders^5,6^.

The present research is devoted to the remote evaluation of swallowing among adults and especially among infants by modeling sucking–swallowing patterns to determine whether a baby is breastfeeding and, if so, to measuring milk consumption. Once we know how to answer these questions reliably, we could determine if a baby is fed properly or if an external intervention to help the baby’s development is required. Because the sucking–swallowing sequence and composition could indicate the existence of a feeding problem, the remote detection of feeding problems is very important.

### The swallowing volumes

There is a significant difference in average swallowing volumes between adults and infants. The three phases of swallowing among infants were identified as a function of age. The average swallowing volume for the children of 24 to 90 months of age was found to be similar to that for males and females. The average volume of a single swallow of children 24 to 42 months old was 3.8 ± 2.4 ml. The total feeding volume for a newborn on average is approximately 45–90 ml, consumed every 2–3 h. At 2 months, the baby may be taking 120–150 ml every 3–4 h. At 4 months, approximately 120–180 ml and by 6 months, approximately 180–230 ml every 4 to 5 h.

In contrast, the standard bolus size for a single swallowing in adults should be set at approximately 20 ml for women and 25 ml for men, regardless of age. It was also reported that the mean bolus volume for men and women is 25.6 ±8.5 ml during a single swallow and 21.1 ± 8.2 ml during stress^7–9^.

### Methods of testing swallowing

Complex set of motor and sensory nerves are involved in the swallowing process. Any dysfunction in this process causes swallowing difficulty. Swallowing difficulty or in medical term—Dysphagia is condition which describe a disrupted in the swallowing process and impaired in the eating ability. The main risks for dysphagia are neurologic and neuromuscular disorders. Impaired or lost the ability to swallow increase the option to disability and even death^10–13^. For this reason, many studies on the subject were conducted.

Dysphagia can be detected today by a number of methods. One of the methods called video fluoroscopy allows examination of both the structure and function of the organs involved. Dysphagia can be identified by this method, and silent aspiration can be detected. The success of this procedure depends on swallowing of a controlled quantity of radio-opaque material, and a subject must be exposed to radiation that must be brief and cannot be used frequently. Fiberscope endoscopic examination (FEES) of swallowing is another method that allows assessment of the areas surrounding the voice box and opening of the esophagus using a small flexible telescope. As an invasive procedure, it is more acceptable for some adult patients, but it is in essence a cumbersome technique not being adapted for children^14,15^. The electromyography (EMG) method is also used for the examination and has advantages of precision in the identification and accuracy of temporal measurements. However, this method is based on the signals derived from electrodes inserted subcutaneously. While EMG is a valuable research tool, it is not well suited for routine clinical investigation^15^. The cervical auscultation (CA) method contains a sensor (originally a stethoscope) placed on the neck of a subject and either allows listening and/or recording of the acoustic signals. These signals could be visually detected and examined. In an attempt to define the optimal configuration, a variety of sensors have been tested, but the best choice of a sensor, or its combination, is still not clear^15^. Transnasal esophagoscopy (TNE) method uses a camera passing through the nose as a way to examine the esophagus in patients at risk for esophageal cancer and other disorders^13,14^. In addition, currently, the well-known and effective methods for testing throat areas include CT scans and MRI. The methods described above are complicated and require ambulatory investigations and sophisticated equipment, which is justified for complicated medical conditions.

Color image processing is not a common method for swallowing detection. While the speckle-based method based on the detection of skin vibrations seems more reliable.

There are two main methods of evaluating swallowing among infants. Swallowing could be detected by listening to the infant’s throat with a stethoscope. Otherwise, using a precise digital scale before and after feeding could allow us to estimate the feeding volume.

The research presented in this paper is focused on the development of a noninvasive measurement and tracking tool for the analysis and quantification of swallowing in elderly individuals and sucking–swallowing in infants. The research is based on noninvasive analyses of speckle patterns scattered from a subject when his neck is illuminated by a laser beam spot over the course of feeding.

## Theoretical background

### Secondary speckles patterns

**Figure 3:**
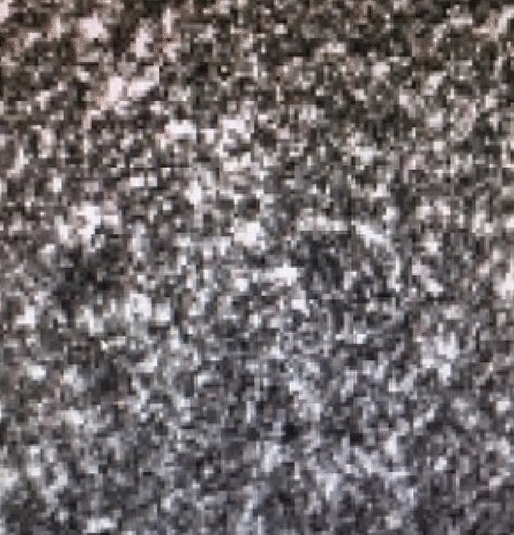
Speckles pattern example.

Speckle pattern analysis has been found by many researchers to be applicable for remote sensing of various biomedical parameters. The analyses were used in a variety of applications: microscopy, imaging, and optical manipulations^16^. When coherent light is reflected from an object, the surface roughness generates a random phase distribution. In this way, self-interfering speckle patterns are generated^16,17^. Speckles pattern example presented in Fig. 3.

In the proposed system configuration, a laser illuminates the neck of a tested subject, and a digital camera captures the reflected speckle patterns. The camera is focused on the far or close field, and the object itself is defocused.

This makes the movement of the object (its vibrations) cause a lateral shift of the speckle pattern. Thanks to the defocusing, the movement of the object creates a situation in which the same speckle pattern is only moving in the transversal plane instead of constantly changing the pattern. This allows extraction of the movement trajectory by tracking the maximal intensity spots which is a very important feature^18^.

For this reason, speckle techniques have been widely used for displacement measurement and vibration analysis. It produces correlation fringes corresponding to the object’s local surface displacements between two exposures and from this determines the magnitude and the direction of the object’s local surface displacement^18,19^.

Zalevsky et al. presented a new technique for remote object vibration measurement based on tracking the temporal changes in both the position and amplitude of the secondary speckle patterns reflected from human skin illuminated by a laser beam^17,19^.

Following the theoretical explanation given by Zalevsky et al.^18^, the system configuration requires defocusing of the imaging camera rather than focusing on the far or close field. This makes the movement of the object (its vibrations) cause a lateral shift of the speckle pattern. Actually, due to defocusing, movement of the object instead of constantly changing the speckle pattern creates a situation where the same speckle pattern is only moving or vibrating in the transversal plane. This is a very important feature since it allows, by tracking the maxima intensity spots, extraction of the movement trajectory. The system allows estimating the 3D trajectory of the object. The skin vibration contains three components: axial, transversal and tilting movement.

The three types of movement are not separated, but it was proven that only tilting movement produces a pure shift of the same speckle pattern, which may easily be detected by spatial pattern correlation. The cross-correlation algorithm takes two adjacent frames and finds the peak location shift measured in pixels.

Our correlation technique is based on the frequency domain image multiplication, following the correlation of the space-frequency attribute of the Fourier transform. This is done in order to reduce the computational complexity of the numerical calculations by the factor of log (N) in each dimension. The two adjacent in-time images are correlated in the frequency domain by multiplication, and later the multiplication product is back-transformed to the image space. The correlation peak is obtained (similar to the space correlation), and its location is calculated in sub-pixel resolution, using the center-of-mass method of surrounding pixels.

Correlation of two adjacent speckle patterns I and T could be determined as follows: The inner product between the vector version t of T and the vector version w(r, c) of window W(r, c) at position (r, c) in the image I can be presented as follows:

1 $$J\left(r,c\right)= \sum_{u=-h}^{h}\sum_{v=-h}^{h}I\left(r+u,c+v\right)T\left(u,v\right),$$

where J is the resulting output image. For simplicity, the image T and the window W(r, c) are assumed squares with 2h + 1 pixels on their side, so that h is a bit less than half the width of the window^20^^.^

The decorrelation of the speckle patterns depends on the integration time of the camera. The defocused, far-field speckles were correlated, but the spatial correlation was obtained between two adjacent frames only. Since we worked at high frame rate, the effect of decorrelation was minimal and the speckle pattern between two adjacent frames looked almost identical.

We propose to use speckle pattern analyses for tracking movements of the subject’s neck in the course of feeding to estimate the swallowing volume in elderly individuals and feeding dysfunction in infants.

## Methods

### Experimental setup

The setup for the sucking and swallowing measurements consists of three principal components. A green laser at a wavelength of 532 nm illuminated the inspected object neck to generate secondary speckle patterns. Basler acA800-510 um digital camera with focal length of 55 mm, and a computer processing the captured images. The camera delivers 511 FPS at 800 × 600-pixel resolution.

The system overseas the secondary speckle patterns reflected from a neck and tracks the trajectory of its movement over time. The captured videos are analyzed by MATLAB software using a cross-correlation algorithm.

The distance between the laser and the tested individual was approximately 45 cm, and the distance between the camera and the tested individual was 25 cm.

Six participants aged 25 to 73, of whom four were men and two women with no background problems, were tested for simulation and remote detection of sucking and swallowing.

Ethics approval for the study was provided by the institutional review board of Bar-Ilan University. All participants provided informed consent for participation in the study. The experiments were carried out in accordance with relevant guidelines and regulations. The device, although dismantled for lab optimization purposes, is fully laser safe, tissue safe, etc. as was previously obtained from international regulators. The participant informed consent was obtained to publish the image in an online open access publication.

### Swallowing

The swallowing process among adults was conducted with the sip volume varying within 5–30 ml. Each participant consequently swallowed in one-sip the premeasured six portions of water, 5, 10, 15, 20, 25, and 30 ml. The number of repetitions for a particular portion of water was determined on the basis of the preliminary evaluation, considering a confidence level of 95% and a measurement error of 10%. During the preliminary tests, we found that the required number of repetitions was five.

Before any measurement, the participant was holding a portion of water in the mouth and swallowed it in one sip. The size of the illuminated tissue area was approximately 3 mm. The protocol we used to position the laser spot on the throat instructed to direct the laser on the preliminary marked laryngeal prominence. The process of water swallowing was recorded at a rate of 20 frames per second (FPS) for 6 s.

**Figure 4:**
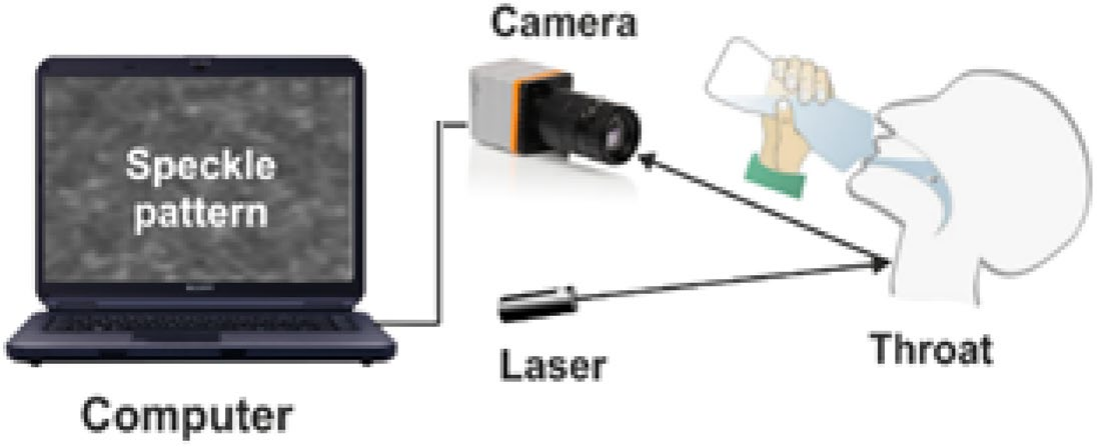
Schematic configuration of the experimental setup.

**Figure 5:**
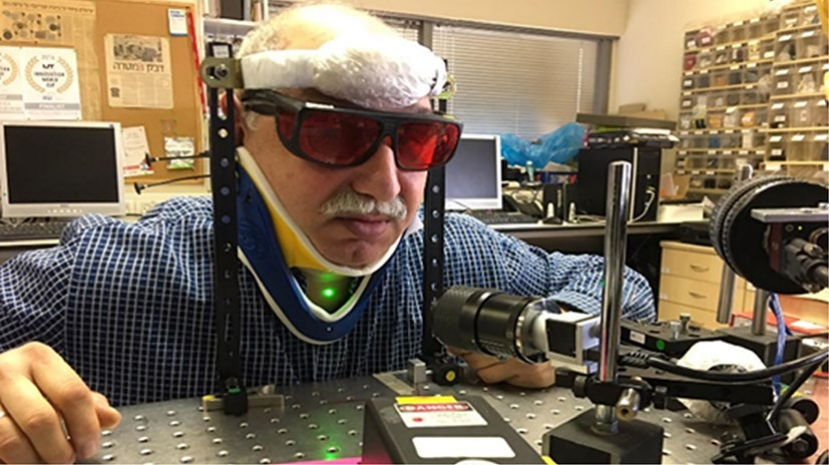
Setup of the system with its optics and the laser illuminating the neck of one subject.

A schematic configuration of the experimental setup is shown in Fig. 4. The setup is presented in Fig. 5.

### Sucking and swallowing

This stage simulated sucking–swallowing among infants. The goal of the experiment was to track sucking swallowing by simulating feeding similar to the feeding of infants. The volumes of water that a subject was sucking and swallowing corresponded to the typical volumes swallowed by an infant. First, the subjects were required to mimic the process of sucking–swallowing in infants. The adult subject received a baby feeding bottle and performed the sucking–swallowing action imitating the process of sucking and swallowing in infants (ratio of 1:1). Then, we simulated the process of improper infant feeding with a sucking–swallowing ratio of 5:1 and above. Each participant sucked and swallowed water from an infant feeding bottle filled in 20 ml of water. Water was sucked and swallowed for 20 s in three ways: ordinary, fast and slow. After evaluating the amount of water consumed and the number of cycles executed during the measurement period, the average volume of water in one sip was calculated.

## Results

### Swallowing

**Figure 6:**
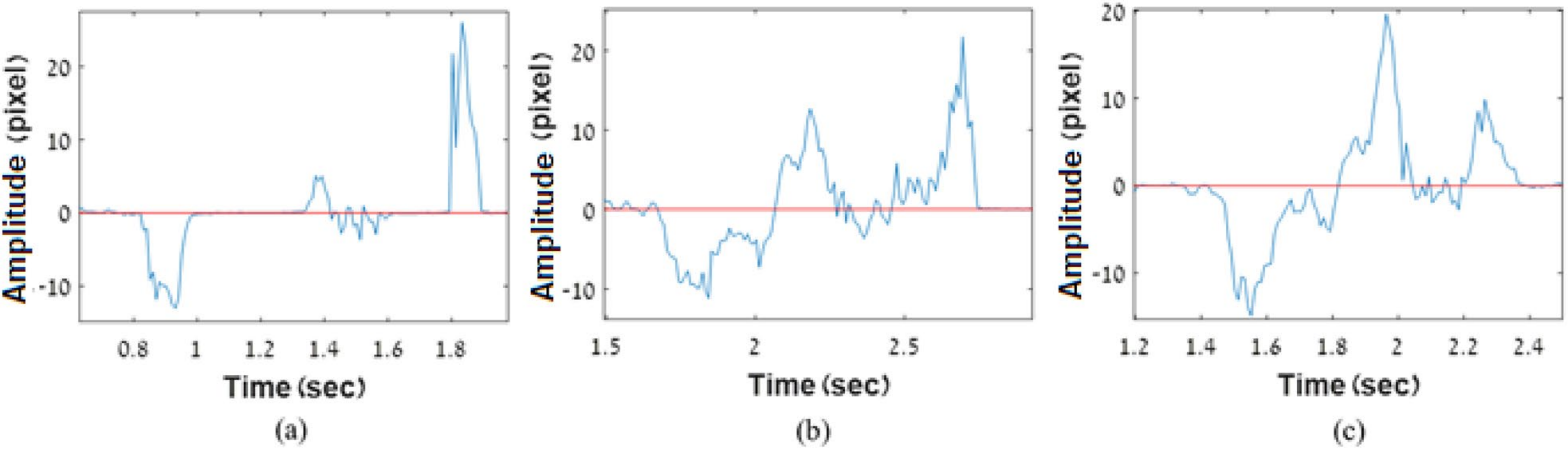
Typical signals for 3 participants.

The following typical signals were recorded during swallowing (see Fig. 6) and processed by a cross-correlation algorithm, analyzing two adjacent frames of the speckle images to find the peak location shift between the frames, measured in number of pixels. The value of the peak shift in pixels is presented in the graph as a function of time. Before swallowing, the average value of the shift is relatively small (approximately 0) because it reflects the noise level only.

It should be mentioned that noise resulted from the inner layers of a skin does not affect the speckle pattern due to the attenuation and scattering of light in tissue media. The speckle pattern is blurred and has less contrast than one reflected from the surface of a skin. Therefore, this noise component of the pattern does not affect the resulted signal significantly.

**Figure 7:**
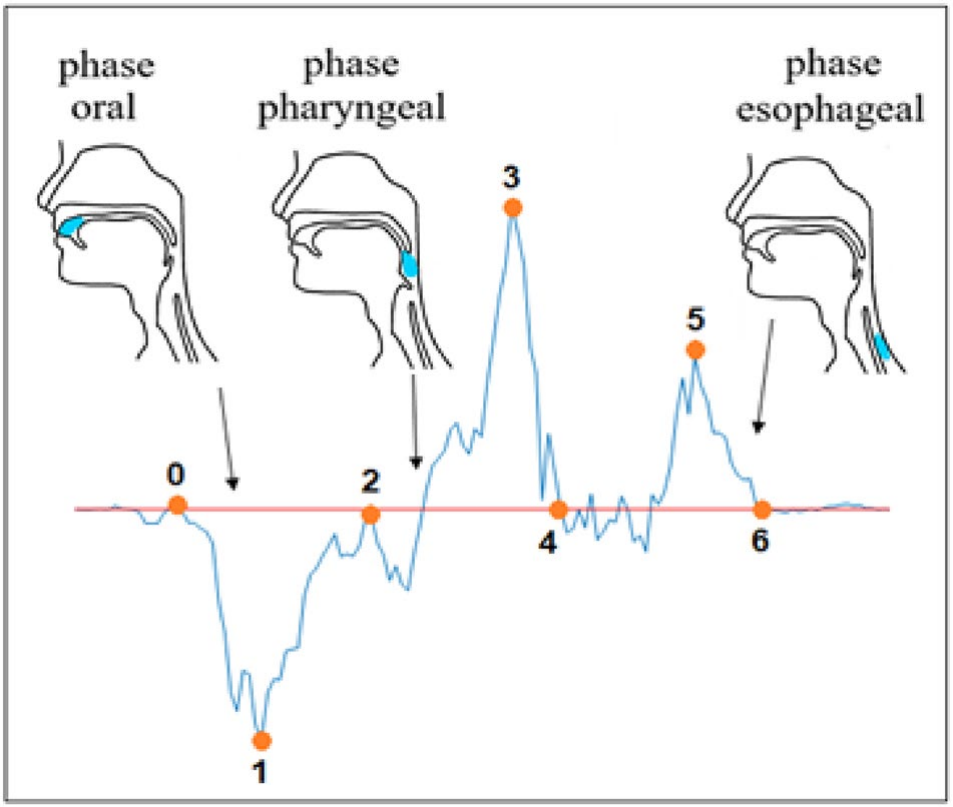
Typical signal: an example of typical signal obtained from one of the subjects during water swallowing with the key points arbitrary selected to analyze the swallowing.

Each recording was subdivided into a number of sections by selecting the key points from 0 to 6, as presented in Fig. 7. For the purpose of the analysis, we denote ${t}_{i}$ as the time when key point i occurred and the interval ${t}_{i}-{t}_{j}$ as ${t}_{ij}$. The signal contains 3 main peaks with a significantly higher amplitude than the rest of the signal. Each peak apparently expresses a different stage of ingestion. The six points were defined manually in an arbitrary way, but they could be automated in the future.

For each peak, we assumed its starting and ending point and assumed the duration of each stage.

- Phase 1—points 0, 1, 2—oral phase.
- Phase 2—points 2, 3, 4—pharyngeal phase.
- Phase 3—points 4, 5, 6—esophageal phase.

The results, containing values $d{t}_{ij}$, representing the three stages of swallowing, were analyzed for each participant. The swallowing cycle parameters $d{t}_{02}, d{t}_{24}, d{t}_{46}$ represent the duration of the oral, pharyngeal and esophageal phases, while $d{t}_{60}$ represents the total duration of the swallowing cycle.

For each amount of water in a sip, the average, standard deviation and variation coefficient were calculated. Correlations between the amount of swallowed water in one sip and the time-related parameters ($d{t}_{ij}$) were calculated for each participant.

Table 1 shows the average $d{t}_{ij}$ (s) for different swallowed volumes (ml) for one participant.

**Table 1:** Average $d{t}_{ij}$ (s) for different swallowed volumes (ml) of water for one participant.

Swallowed volume V (ml)	$d{t}_{31}$	$d{t}_{51}$	$d{t}_{53}$	$d{t}_{60}$	$d{t}_{10}$	$d{t}_{21}$	$d{t}_{54}$	$d{t}_{65}$	Phase 1 $d{t}_{20}$	Phase 2 $d{t}_{42}$	Phase 3 $d{t}_{64}$
5	0.29	0.50	0.21	0.74	0.14	0.15	0.12	0.11	0.29	0.22	0.23
10	0.46	0.63	0.17	0.84	0.09	0.11	0.14	0.12	0.20	0.38	0.26
15	0.42	0.72	0.29	0.87	0.07	0.05	0.23	0.08	0.12	0.44	0.31
20	0.44	0.86	0.43	1.33	0.13	0.14	0.32	0.34	0.27	0.40	0.66
25	0.39	0.78	0.40	1.26	0.07	0.06	0.33	0.41	0.13	0.39	0.74
30	0.30	0.58	0.29	1.41	0.07	0.07	0.15	0.76	0.14	0.36	0.91

Correlation coefficients between the swallowed volumes in one sip and the time-related parameters ($d{t}_{ij}$) were also calculated. Strong correlations were obtained for ${t}_{60}$, $d{t}_{65}$ and $d{t}_{64}$, with correlation coefficients with swallowed volumes of 0.94, 0.9, and 0.96, respectively.

The three mentioned parameters are also cross correlated and therefore belong to the same classification factor. Therefore, only one parameter $d{t}_{60}$—the total swallowing duration—was selected for further analyses. The total swallowing duration had the lowest variation, and five repetitions of the experiments were sufficient for the established precision. The testing results were similar for the rest of the tested subjects. Due to the abovementioned factors, we concluded that the most solid feature related to swallowing volume, which is common for all participants, is the total swallowing duration, $d{t}_{60}$.

Table 2 contains the range of correlation coefficients between the swallowed volume and $d{t}_{ij}$ for all participants.

**Table 2:** Range of correlation coefficients between the swallowed volume and $d{t}_{ij}$.

Time related parameter of the swallowing cycle	Correlation range between the swallowed volume and $d{t}_{ij}$ for all participants
$d{t}_{31}$	0.11–0.86
$d{t}_{51}$	0.42–0.97
$d{t}_{53}$	0.21–0.98
$d{t}_{65}$	0.06–0.90
$d{t}_{10}$	0.09–0.63
$d{t}_{21}$	0.24–0.83
$d{t}_{54}$	0.08–0.69
$d{t}_{20}$ (Phase 1)	0.01–0.88
$d{t}_{42}$ (Phase2)	0.49–0.98
$d{t}_{64}$ (Phase 3)	0.08–0.96
$d{t}_{60}$	**0.87–0.99**

Significance value is given in bold.

Table 2 shows that the $d{t}_{60}$ feature (swallowing duration) yields a strong and stable correlation with the swallowing volume for all participants: values ranging from 0.87 to 0.99.

**Figure 8:**
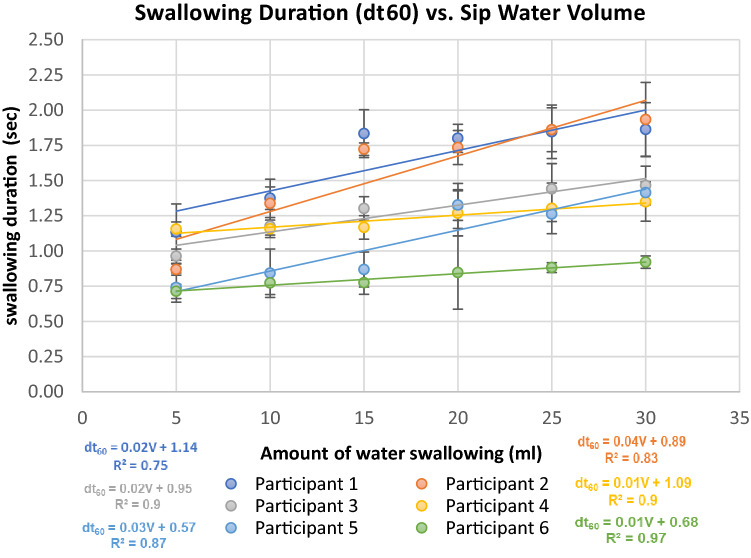
Swallowing duration vs. water sip volume for each participant.

Linear regression between swallowing duration $d{t}_{60}$ and water sip volume for each participant is presented in Fig. 8; for example, under a swallowing volume of 5 ml, the sip duration varies within 0.8–1.4 s, while for a volume of 30 ml, the sip duration varies between 0.9 and 2.1 s.

The parameter $d{t}_{60}$ presents the total duration of swallowing. The total duration could be measured more precisely by observing the noise level compared to other cycle elements $d{t}_{\mathrm{ij}}$, which could be identified in an arbitrary way (Fig. 6). The result is also logic due to the assumption that swallowing of large volumes takes longer period.

**Figure 9:**
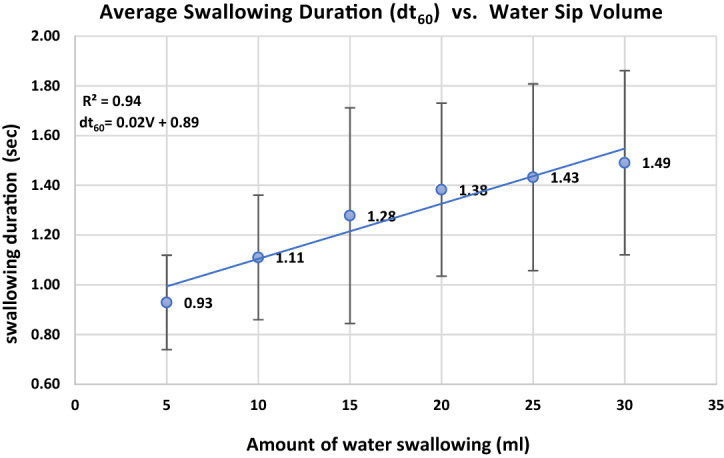
Average swallowing duration vs. sip volume for all participants.

Figure 9 represents the relation between swallowing duration and sip volume for all participants. The average swallowing duration vs sip volume varied within 0.9–1.5 s and showed a strong linear correlation (R = 0.94).

The results show a strong correlation between the total duration of swallowing $(d{t}_{60})$ starting in the oral phase and ending in the esophageal phase and the amount of water in a sip.

### Sucking and swallowing

**Figure 10:**
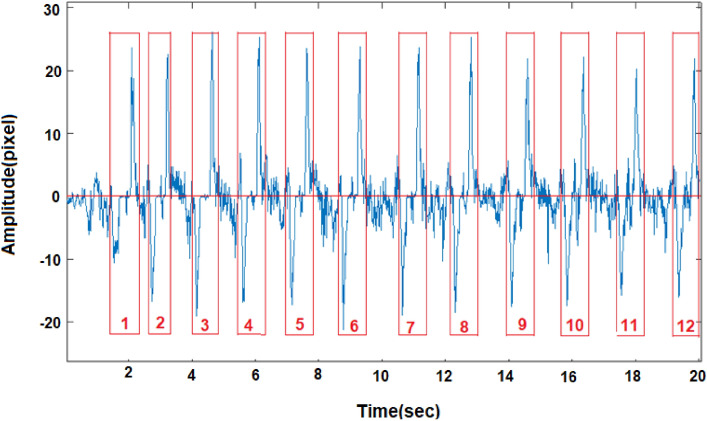
A typical graph of rhythmic water drinking containing 12 sucking–swallowing cycles.

The process of feeding by infants comprises sucking and swallowing. We simulated the mentioned process by requesting the participants to suck and swallow relatively small amounts of water from a baby feeding bottle. The process was recorded, and the video files were processed by the cross-correlation algorithm. A typical graph of the consequent water sucking–swallowing (1:1) is presented in Fig. 10. The swallowing phases consist of high amplitude (in red blocks) in contrast to the sucking phase in between, having a low amplitude.

**Figure 11:**
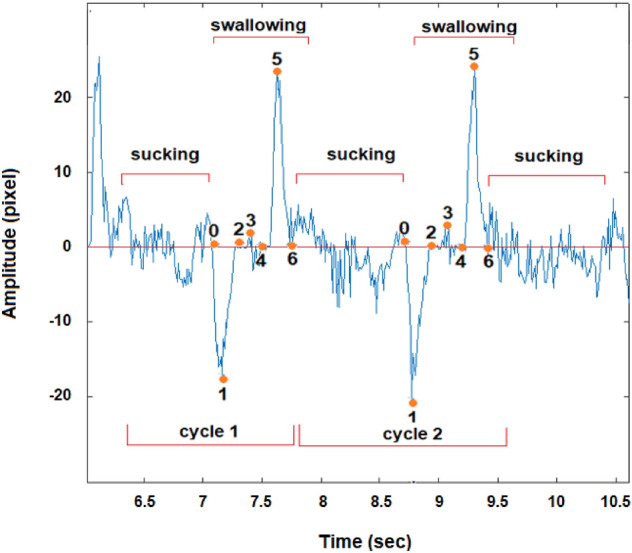
A typical graph of a healthy drinking process. The process combines cycles of sucking and swallowing in the ratio of 1:1.

Figure 11 presents a partial graph of the same process, detailing the sucking and swallowing phases during rhythmic sucking–swallowing cycles (1:1). The key points of swallowing are specified in “Swallowing” section.

**Figure 12:**
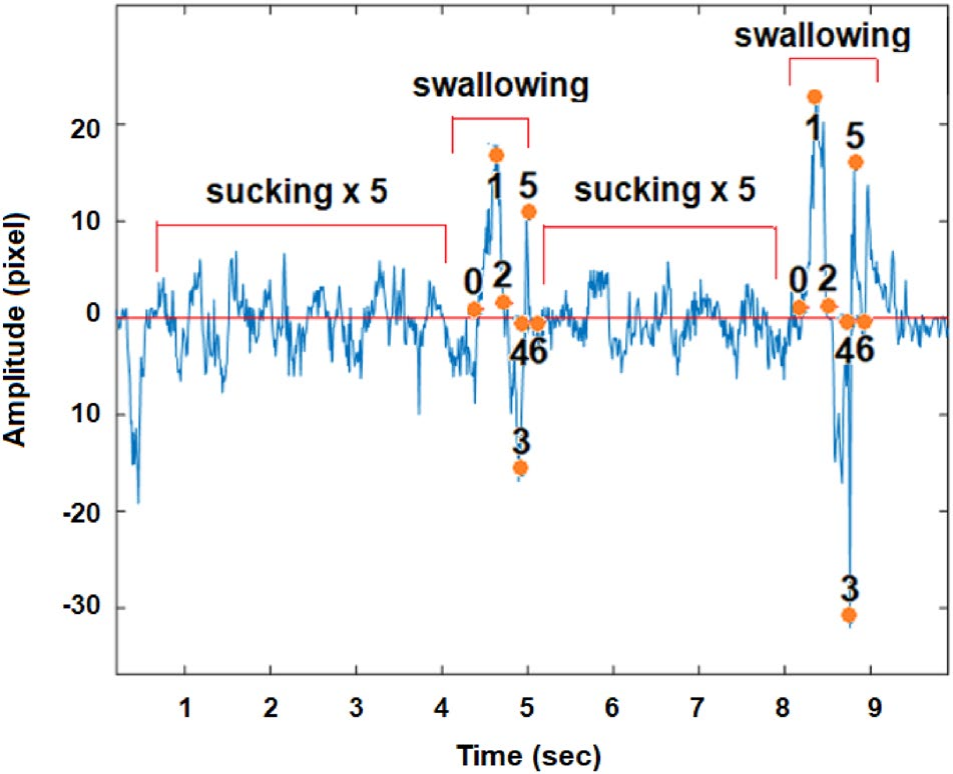
A typical graph of a problematic drinking process. The process combines sucking and swallowing in the ratio of 5:1.

Figure 12 shows the simulation of a problematic sucking–swallowing process. The process combines sucking and swallowing at a ratio of 5:1. The key points of the swallowing were specified.

Figures 11 and 12 show that the feeding simulation by our new noninvasive method allows identification of a feeding disorder and could be applied in infants. As we already mentioned, suck-to-swallow ratios of 2:1 and/or 1:1 are normal ratios, but ratios of 5:1 or higher are called “flutter suck” or “nonnutritive suck” and are associated with piston movements of the jaw and poor weight gain in the infant. To determine the relation between the sucking–swallowing volume and feeding cycle duration, we measured the amount of water consumed during the known number of feeding cycles. Each participant sucked and swallowed water from the infant feeding bottle for 20 s in three ways: ordinary, fast and slow.

The ‘average cycle duration’ was calculated by evaluating the number of cycles occurring during the test period.

Table 3 shows a strong correlation between the water volume in one suck and cycle duration for all participants (r = 0.88 to 0.99).

**Table 3:** Parameters of the sucking–swallowing cycle duration and correlation between the water volume in one suck and duration of the cycle (s).

Participant	Average water volume in one cycle, V (ml)	Number of cycles	Average cycle duration	Std cycle duration	Variation coefficient, (%) cycle duration	Correlation coefficient between V and cycle duration
1	1.25	8	1.02	0.03	3	
	3	4	2.14	0.27	12	
	3.2	5	1.9	0.11	6	
						0.95
2	1.25	3	2.06	0.04	2	
	1.67	6	3.04	0.32	11	
	1.86	7	2.86	0.36	13	
						0.88
3	0.88	5	1.26	0.22	18	
	1.75	3	2.42	0.55	23	
	2.14	3	2.77	0.62	22	
						0.99
4	0.7	10	1.92	0.15	8	
	2	7	2.59	0.21	8	
	3.75	4	3.63	0.44	12	
						0.99
5	0.42	11	1.59	0.23	14	
	1.43	6	2.65	0.12	5	
	2.5	3	3.19	0.34	11	
						0.99
6	0.2	10	1.75	0.39	22	
	0.5	10	2.18	0.41	19	
	1.14	7	2.8	0.25	9	
						0.99

**Figure 13:**
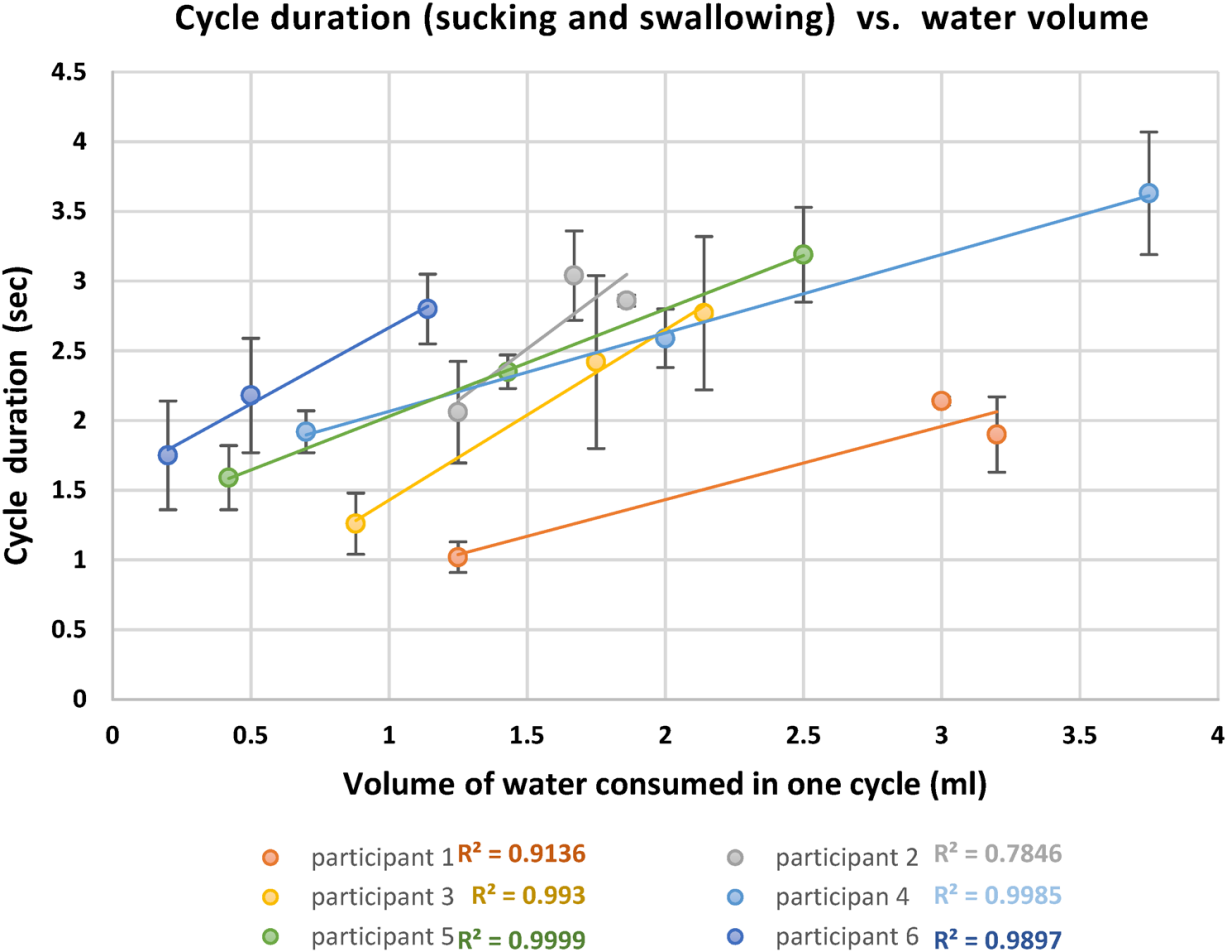
Cycle duration (sucking and swallowing) vs. water volume in a sip for all participants.

Figure 13 shows the linear regression between the sucking–swallowing cycle duration and water volume in one sip for each participant. Each individual has a specific sucking–swallowing curve, and the variation in the sucking–swallowing duration between the participants is significant.

Therefore, monitoring swallowing disorders in elderly individuals requires preliminary testing and calibration for evaluation of swallowing volumes, while identification of dysphagia could be done by analyzing the swallowing graph structure and the proportion between the three swallowing stages. Undoubtedly, additional investigation involving participants with swallowing disorders is required.

In relation to the infants, swallowing disorders could be identified by observing the proportion between sucking and swallowing, combined with measurement of the feeding volume and duration.

We also analyzed sacking–swallowing in the frequency domain and haven’t found specific relation between the process and frequency distribution.

## Conclusions

The developed new method for remote detection and quantification of water sucking and swallowing is based on the analyses of secondary speckle patterns reflected from the subject’s neck. A laser beam illuminates the neck of the tested subject, and a digital camera captures the reflected speckle patterns.

The obtained recordings were analyzed in the temporal and frequency domains to distinguish between the swallowing phases and to determine the relation between the volume of water in a sip and the parameters of the swallowing cycle. Three swallowing phases (oral, pharyngeal and esophageal) could be identified, and their duration was evaluated.

To determine the relation between the volume of water in a sip and the time-related parameters, the swallowing cycle was subdivided into six intervals $d{t}_{ij}$, and the correlation between $d{t}_{ij}$ and the volume of water in a sip was calculated. It was found that the total swallowing duration (containing the three phases) had a high correlation with the volume of water in a sip for all tested participants. Such phenomena could be explained by the physical dependence between the swallowing volume and its duration, while other cycle elements are identified manually in an arbitrary way.

Simulation of feeding in infants shows that sucking and swallowing phases could be clearly identified. The sucking phase has a relatively low amplitude, and the swallowing phase shows a higher amplitude and contains the three phases of swallowing (oral, pharyngeal and esophageal). The method shows the possibility of remote quantification and sensing of feeding disorders in infants by evaluating the feeding cycle duration and the proportion between the sucking and swallowing sequences in the course of feeding. The method allows the determination of the relation between the volume of water in a sip and the parameters of the sucking and swallowing cycle.

The promising results obtained along with the inherent benefits of the system (noninvasive, simple signal analysis) are the basis for future investigations involving infants to quantify and control the feeding process.

## Data Availability

The data generated to support the findings of this study are available from the corresponding author upon reasonable request.
